# Hemodynamic effects of short-term hyperoxia after coronary artery bypass grafting

**DOI:** 10.1186/s13613-017-0246-9

**Published:** 2017-02-23

**Authors:** Hendrik J. F. Helmerhorst, Rob B. P. de Wilde, Dae Hyun Lee, Meindert Palmen, Jos R. C. Jansen, David J. van Westerloo, Evert de Jonge

**Affiliations:** 1 Department of Intensive Care Medicine, Leiden University Medical Center, Post Box 9600, Leiden, 2300 RC The Netherlands; 2 Department of Anesthesiology, Leiden University Medical Center, Leiden, The Netherlands; 3 Laboratory of Experimental Intensive Care and Anesthesiology, Academic Medical Center, Amsterdam, The Netherlands; 4 Department of Nephrology, Einthoven Laboratory for Vascular Medicine, Leiden University Medical Center, Leiden, The Netherlands; 5 Department of Cardiothoracic Surgery, Leiden University Medical Center, Leiden, The Netherlands

**Keywords:** Hyperoxia, Arterial oxygenation, Hemodynamics, Microcirculation, Cerebral blood flow velocity, Intensive care unit, Thoracic surgery

## Abstract

**Background:**

Although oxygen is generally administered in a liberal manner in the perioperative setting, the effects of oxygen administration on dynamic cardiovascular parameters, filling status and cerebral perfusion have not been fully unraveled. Our aim was to study the acute hemodynamic and microcirculatory changes before, during and after arterial hyperoxia in mechanically ventilated patients after coronary artery bypass grafting (CABG) surgery.

**Methods:**

This was a single-center physiological study in a tertiary care ICU in the Netherlands. Twenty-two patients scheduled for ICU admission after elective CABG were enrolled in the study between September 2014 and September 2015.

In the ICU, patients were exposed to a fraction of inspired oxygen (FiO_2_) of 90% allowing a 15-min wash-in period. Various hemodynamic parameters were measured using direct pressure signals and continuous arterial waveform analysis at three sequential time points: before, during and after hyperoxia.

**Results:**

During a 15-min exposure to a fraction of inspired oxygen (FiO_2_) of 90%, the partial pressure of arterial oxygen (PaO_2_) and arterial oxygen saturation (SaO_2_) were significantly higher. The systemic resistance increased (*P* < 0.0001), without altering the heart rate. Stroke volume variation and pulse pressure variation decreased slightly. The cardiac output did not significantly decrease (*P* = 0.08). Mean systemic filling pressure and arterial critical closing pressure increased (*P* < 0.01), whereas the percentage of perfused microcirculatory vessels decreased (*P* < 0.01). Other microcirculatory parameters and cerebral blood flow velocity showed only slight changes.

**Conclusions:**

We found that short-term hyperoxia affects hemodynamics in ICU patients after CABG. This was translated in several changes in central circulatory variables, but had only slight effects on cardiac output, cerebral blood flow and the microcirculation.

*Clinical trial registration* Netherlands Trial Register: NTR5064

## Background

During and after coronary artery bypass grafting (CABG), patients are supported with mechanical ventilation and supplemental oxygen. Despite its lifesaving characteristics and key role in the treatment of vasodilatory shock, oxygen therapy may harbor considerable risks given the relationship between prolonged hyperoxia, lung injury and adverse outcome [1–5]. The effects of supplemental oxygen may be even more pertinent during cardiovascular events considering the direct effects of high arterial oxygen concentrations on the vascular tone [6, 7]. Arterial hyperoxia has the potential to alter hemodynamics and has been associated with adverse outcomes and mortality after cardiac arrest, myocardial infarction, stroke, brain injury and during mechanical ventilation [8–14], yet not during cardiopulmonary bypass [15, 16]. It is well established that high oxygen concentrations induce vasoconstriction and increase the resistance of the systemic circulation. However, the effects on vital parameters may be diverse and the venous and arterial aspects of the circulation have not been clearly distinguished in previous studies. Furthermore, the microcirculation may react differently than systemic hemodynamics [17].

Achieving hemodynamic stabilization is an important clinical prerequisite for early extubation and dismissal from the intensive care unit (ICU) after cardiothoracic surgery. Any intervention that influences hemodynamics and blood flow to the bypassed myocardial territories may impact functional recovery and requires optimal fine-tuning to achieve the best outcome. Although oxygen is generally administered in a liberal manner in the perioperative setting, the unraveling of the effects of oxygen administration on dynamic cardiovascular parameters, filling status and cerebral perfusion may provide novel insights in the pathophysiological mechanisms involved in hyperoxic exposure. Our aim was to study the acute hemodynamic and microcirculatory changes during increased oxygen supply in mechanically ventilated ICU patients after CABG surgery.

## Methods

### Participants

Adult patients with symptomatic coronary artery disease without recent myocardial infarction scheduled for ICU admission after coronary artery bypass surgery were screened for eligibility. Patients with congestive heart failure, severe arrhythmias, intracardiac shunts, extensive peripheral arterial occlusive disease, symptomatic pulmonary disease, aortic aneurysm and/or significant valvular disease were not considered for inclusion. Patients with signs of severe hemodynamic instability (e.g., rapid changes in vascular resistance, use of inotropic agents) during ICU admission were excluded. Study approval was granted by the local medical ethics committee (LUMC P14.046), and all patients signed informed consent. The study was registered with the Netherlands Trial Register, number NTR5064, registration date February 2015.

### Measurements

Anesthesia during surgery was maintained with propofol and sufentanil. Ventilation was adjusted to achieve normocapnia. FiO_2_ was 0.4, and a positive end-expiratory pressure of 5 cm H_2_O was applied. Directly after surgery, patients were admitted to the ICU and received standard postoperative care. Continuous infusion of propofol and sufentanil was maintained for all patients, and no bolus medications (fluids, vasoactive or sedative agents) were administered.

Mean arterial blood pressure (MAP) was measured via a 20-G radial arterial catheter inserted by Seldinger technique. Central venous pressure (CVP) was measured with a central venous catheter inserted in the right internal jugular vein (MultiCath venous catheter, Vigon GmbH & Co, Aachen, Germany). Pressure transducers (PX600F, Edwards Lifesciences) for the arterial and central venous signals were referenced to the intersection of the anterior axillary line and the fifth intercostal space. The airway pressure was measured at the entrance of the endotracheal tube and balanced at zero level against ambient air. Standard electrocardiogram leads were used to monitor heart rate. Body temperature was measured using a rectal temperature probe.

Beat-to-beat values of cardiac output (CO), stroke volume, stroke volume variation (SVV), pulse pressure variation (PPV) and heart rate (HR) were obtained by Modelflow using continuous arterial waveform analysis as previously described [18, 19]. Hemodynamics were also monitored by the LiDCO*plus* monitor (LiDCO Group Plc., London, UK).

Before starting the protocol, the mechanical ventilation in volume-controlled mode was switched to airway pressure release ventilation (APRV), with settings adjusted to achieve the same minute ventilation, which allows for external control of the ventilator (Evita 4, Dräger AG, Lübeck, Germany). A computer program was used to control the ventilator as described previously [20]. During the study interval, all patients were hemodynamically stable and ventilator settings, sedation and vasoactive therapy remained unchanged.

At least three videos of ten sequences (40 frames each) visualizing different sites of the sublingual microcirculation were recorded per patient per time point by the same dedicated researcher using sidestream dark field (SDF) imaging with the MicroScan Video Microscope (MicroVision Medical BV, Amsterdam, The Netherlands). The three best quality videos from representative multiple site imaging were analyzed, and calculated parameters were averaged. Previously suggested key points for optimal image acquisition were considered, and maximal efforts were undertaken to avoid pressure artifacts and eliminate secretions [21]. SDF imaging data were recorded and analyzed using real-time quality feedback on adequate focus, contrast and stability with GlycoCheck (GlycoCheck BV, Maastricht, The Netherlands), as described previously [22]. The GlycoCheck software automatically calculates the perfused boundary region (PBR), which is a previously validated dimension of the permeable part of the endothelial glycocalyx that does allow red blood cell penetration [23, 24]. The red blood cell (RBC) filling percentage is calculated an estimate for microvascular perfusion. Recorded videos were also imported for offline analysis in Automated Vascular Analysis (AVA) software 4.1 (MicroVision Medical BV). The software automatically separates outcome parameters for large (mostly venules) or small (mostly capillaries) vessels using a diameter cutoff value of 20 μm. Total vessel density (TVD), perfused vessel density (PVD), valid vessel density (VVD) and De Backer Score were calculated as measures of microvascular vessel density; the percentage of perfused vessels (%PV) was calculated as the number of vessels continuously perfused divided by the total number of vessels of the same type. The heterogeneity index was defined as the difference between maximal and minimal proportions of perfused vessels evaluated at each visualized area divided by the mean value of the areas [25].

Blood flow velocity (BFV) in the right middle cerebral artery (MCA) was measured at an insonation depth of 50–52 mm by transcranial Doppler (TCD) monitoring using a Pioneer TC 4040. When the optimal TCD signal was achieved, a 2-MHz TCD transducer probe was fixed over the temporal window using an adjustable headset (Marc 500, Spencer Technologies, Nicolet Biomedical).

### Experimental procedure

Approximately one hour after ICU admission the experimental procedures were initiated. All measurements were performed with patients in supine position at three sequential time points: pre-intervention, during intervention and post-intervention. Before the intervention (T1), FiO_2_ was titrated to a level targeting a partial pressure of arterial oxygen (PaO_2_) between 67.5 mmHg (9 kPa) and 82.5 mmHg (11 kPa) and a complete set of hemodynamic measurements was performed. The intervention (T2) commenced by increasing the FiO_2_ to 0.9, and after a 15-min wash-in period, all hemodynamic measurements were repeated. Thereafter (T3), the FiO_2_ was decreased by targeting baseline PaO_2_ levels, and after 15-min wash-out period, the final control measurements were completed. Before, during and after the intervention, arterial blood gas samples were analyzed to determine arterial oxygenation.

Four 12-second inspiratory hold maneuvers were applied using ventilator plateau pressures of 5, 15, 25 and 35 cm H_2_O as previously reported [20]. Each successive inspiratory hold was performed when the initial hemodynamic steady state was reestablished. When the plateau pressure increases, CVP increases concomitantly, whereas CO and MAP decrease with a short delay, reaching a steady state at 7–10 s after inflation. From these steady state measurements, a venous return curve was constructed by fitting a linear regression line through four values of CVP and CO. The extrapolated value at zero flow is the mean systemic filling pressure (*P*_msf_). Similarly, the ventricular output curve was fitted through the values of MAP and CO, where the regression line crosses the zero flow intercept at the critical closing pressure (*P*_cc_) [19].

The resistance of the systemic circulation (*R*_sys_) was calculated as the ratio of the pressure difference between MAP and mean CVP, and CO. The resistance at the arterial and venous side of the circulation was also separately calculated as resistance for ventricular output *R*_vo_ = (MAP − *P*_cc_)/CO and resistance for venous return *R*_vr_ = (*P*_msf_ − CVP)/CO [26].

### Statistical analysis

As this was an exploratory physiological intervention study studying multiple hemodynamic parameters, we did not specifically rely on sample size calculation for one single outcome.

The intervention (T2, *hyperoxia*) and post-intervention measurements (T3, *normoxia*) were compared to baseline (T1, *normoxia*) measurements, using a paired *t* tests or Wilcoxon signed-rank test, depending on the underlying distribution.

Multivariate linear mixed models with random effects per patient were used to compare the exposure (T2) with the non-exposure (T1 and T3) measurements, to account for within-subject correlation and were adjusted for age, temperature, the administered dose of propofol and norepinephrine, and the achieved levels of arterial carbon dioxide (PaCO_2_) and hemoglobin (Hb).

To account for multiple testing, the indicated levels of statistical significance were lowered to 0.01. All statistical analyses were conducted using R version 3.2.1 (R Foundation for Statistical Computing, Vienna, Austria).

## Results

Patients were screened for eligibility from September 2014 until September 2015. Four patients were excluded due to severe postoperative hemodynamic or respiratory instability in the ICU. Baseline characteristics of the twenty-two included patients are listed in Table 1. All participating patients were free of surgical complications, fully recovered from anesthesia within 8 h after surgery and were discharged from the ICU on the first postoperative day. During the experimental procedure, all patients received a glucose 2.5% in 0.45% saline solution at 84 ml/h, propofol (range 200–400 mg h^−1^) and sufentanil (range 5–25 µg h^−1^). Two patients additionally received norepinephrine (0.02 and 0.04 µg kg^−1^ min^−1^) at a constant rate in order to keep the blood pressure in a similar range (MAP higher than 65 mmHg) as the other included patients during the experimental procedure. This was accounted for in the multivariate linear mixed model, and excluding these patients did not materially change the magnitude or direction of our univariate findings.

**Table 1:** Patient characteristics

Characteristics	All patients (*n* = 22)
Descriptive characteristics	
Age (year)	63 (59–66)
Male/female (*n*)	17/5
BMI (kg/m^2^)	26 (25–29)
Body temperature (°C)	37 (36–37)
APACHE IV	40 (33–61)
SAPS II	28 (24–32)
Surgical characteristics	
Perfusion time (min)	105 (91–121)
Clamp time (min)	73 (63–82)
ICU ventilator settings	
*P*_insp_ (cm H_2_O)	18 (16–19)
*V*_T_ (ml)	585 (484–650)
PEEP (cm H_2_O)	5 (5–5)
Respiratory rate (breaths min^−1^)	12 (12–14)
ICU medication	
Propofol (mg h^−1^)	250 (200–288)
Sufentanil (mg h^−1^)	10 (6–10)
Norepinephrine (µg kg^−1^ min^−1^)	0 (0–0), range 0–0.04

Data are medians (interquartile range), unless stated otherwise

*BMI* body mass index, *APACHE* Acute Physiology and Chronic Health Evaluation Score, *SAPS* Simplified Acute Physiology Score, *P*_*insp*_ inspiratory pressure, *V*_*T*_ tidal volume, *PEEP* positive end-expiratory pressure

### Arterial blood gas parameters

Arterial blood gas values at the three different time points are shown in Table 2. PaO_2_ levels pre- and post-hyperoxia matched well with the targeted levels. Also, pre- and post-hyperoxia arterial oxygen saturation (SaO_2_) was similar. During hyperoxia PaO_2_ and SaO_2_ were significantly higher.

**Table 2:** Variables of arterial blood gas analyses during different time periods

Variable	T1	T2	T3
	Pre	Hyperoxia	Post
FiO_2_ (%)	25 (21–30)	90 (90–90)	21 (21–25)
Arterial blood gas analyses^a^			
SaO_2_ (%)	94.9 (1.9)	99.0 (0.3)***	95.7 (1.8)
PaO_2_ (mmHg)	83.5 (12.2)	390.2 (93.2)***	87.8 (21.5)
PaCO_2_ (mmHg)	39.8 (8.1)	36.0 (7.9)**	34.5 (8.7)***
Hb (mmol L^−1^)	7.2 (0.8)	7.4 (0.7)	7.4 (0.8)
Ht (L L^−1^)	0.34 (0.04)	0.35 (0.03)	0.35 (0.04)
Glucose (mmol L^−1^)	7.5 (1.6)	7.4 (1.7)	7.7 (1.8)
Lactate (mmol L^−1^)	1.25 (0.38)	1.20 (0.40)	1.25 (0.34)

Data are means (SD). For FiO_2_, medians (interquartile range) are provided

*FiO*_*2*_ fraction of inspired oxygen, *SaO*_*2*_ arterial oxygen saturation, *PaO*_*2*_ partial pressure of arterial oxygen, *PaCO*_*2*_ partial pressure of arterial carbon dioxide, *Hb* hemoglobin, *Ht* hematocrit

* *P* < 0.01; ** *P* < 0.001; *** *P* < 0.0001 for paired comparison between indicated outcome and baseline (T1)

^a^Arterial blood gas samples analyzed prior to the start of hemodynamic measurement

PaCO_2_ decreased over time, whereas hemoglobin, hematocrit, glucose and lactate levels did not change.

### Hemodynamic parameters

Hemodynamic values at the three different time points are shown in Table 3. After starting the intervention with 90% oxygen supply, *R*_sys_ increased (*P* < 0.0001), without altering the heart rate. SVV and PPV decreased slightly. CO did not significantly decrease (*P* = 0.08).

**Table 3:** Crude hemodynamic measurements during different time periods and adjusted change in estimate with hyperoxic ventilation

Hemodynamic variables	T1	T2	T3	Hyperoxia vs. normoxia	*P* value
	Pre	Hyperoxia	Post	Adjusted change in estimate (95% CI)	
Central circulatory variables^a^					
MAP (mmHg)	77 (11)	85 (11)***	78 (11)	6.76 (3.88; 9.63)	<0.0001
CVP (mmHg)	9.1 (1.7)	9.6 (1.7)	9.3 (1.6)	0.35 (0.11; 0.60)	0.01
HR (beats min^−1^)	84 (14)	82 (14)	83 (15)	−0.55 (−3.05; 2.06)	0.68
Calculated variables					
CO *Modelflow* (L min^−1^)^b^	5.12 (1.04)	4.97 (1.13)	4.98 (1.18)	−0.08 (−0.27; 0.11)	0.41
SVV (%)^b^	13.6 (9.3)	13.2 (6.9)	15.3 (7.4)	−1.76 (−3.38; −0.03)	0.05
PPV (%)^b^	15.6 (10.3)	15.1 (7.6)	16.6 (4.9)	−1.30 (−2.99; 0.49)	0.16
CO *LiDCOplus* (L min^−1^)^c^	4.80 (1.10)	4.62 (1.10)	4.79 (1.27)	−0.12 (−0.40; 0.08)	0.21
Derived parameters^d^					
*R*_sys_ (mmHg min L^−1^)	13.4 (4.9)	15.3 (5.9)***	13.6 (5.1)	1.82 (0.96; 2.67)	<0.001
*P*_vr_ (mmHg)	11.7 (3.3)	13.5 (3.5)*	12.1 (2.8)	1.47 (0.61; 2.37)	<0.01
*R*_vr_ (mmHg min L^−1^)	2.4 (0.8)	2.8 (1.0)**	2.5 (0.8)	0.39 (0.21; 0.58)	<0.001
Slope_vrc_ (L min^−1^ mmHg^−1^)	−0.46 (0.16)	−0.38 (0.13)**	−0.44 (0.15)	0.07 (0.03; 0.10)	<0.001
*P*_msf_ (mmHg)	20.8 (3.5)	23.1 (4.0)*	21.4 (2.9)	1.90 (0.95; 2.93)	<0.001
*R*_vo_ (mmHg min L^−1^)	7.9 (3.2)	7.9 (4.3)	7.7 (1.9)	−0.09 (−1.17; 1.03)	0.87
Slope_voc_ (L min^−1^ mmHg^−1^)	0.13 (0.05)	0.15 (0.11)	0.14 (0.04)	0.01 (−0.03; 0.05)	0.62
*P*_cc_ (mmHg)	38.8 (9.8)	47.9 (15.1)*	40.9 (8.9)	8.55 (4.13; 12.68)	<0.001
Cerebral blood flow^e^					
BFV_mca_ (cm s^−1^)	34.6 (10.6)	32.3 (10.3)	33.6 (11.5)	−1.42 (−3.80; 1.01)	0.26
Pulsatility index	0.95	0.96	1.0	−0.03 (−0.07; 0.01)	0.17
Resistance index	0.57	0.57	0.58	0 (−0.01; 0.01)	0.66
Microcirculation^f^					
RBC filling (%)	72.3 (4.4)	71.1 (5.0)	72.9 (5.1)	−1.87 (−3.29; −0.34)	0.02
PBR (µm)	2.1 (0.2)	2.2 (0.2)	2.1 (0.2)	0.05 (−0.03; 0.12)	0.21
TVD (mm/mm^2^)	12.2 (3.5)	12.7 (3.5)	12.0 (3.4)	0.35 (−0.96; 2.08)	0.66
PVD (mm/mm^2^)	12.0 (3.7)	12.1 (2.9)	12.0 (3.1)	−0.03 (−1.50; 1.70)	0.97
VVD (µm/mm^2^)	712 (117)	672 (130)	699 (93)	−37.29 (−80.97; 8.01)	0.11
De Backer Score (n/mm)	14.2 (1.2)	15.1 (1.6)	14.4 (1.6)	0.86 (0.23; 1.51)	0.01
PV (%)	99.6 (1.3)	93.2 (8.3)*	97.4 (6.1)	−4.72 (−7.64; −2.00)	<0.01
	100 (IQR 99–100)	98 (IQR 85–100)	100 (IQR 99–100)	–	–
Heterogeneity index (%)	13 [9–14]	22 [21–23]***	21 [15–21]***	–	–

Change in estimate (95% CI) with intervention (hyperoxia) in reference to normoxia periods from linear mixed model adjusted for age, temperature, Hb, PaCO_2_, norepinephrine dose and propofol dose. *P* value calculated using *t* tests with Satterthwaite approximations to degrees of freedom

Data are means (SD). For PV (%), medians (interquartile range) are provided

*MAP* mean arterial pressure, *CVP* central venous pressure, *HR* heart rate, *CO* cardiac output, *SVV* stroke volume variation, *PPV* pulse pressure variation, *R*_*sys*_ resistance of the systemic circulation, *P*_*vr*_ pressure difference between *P*_msf_ and *P*_cv_, *R*_*vr*_ resistance for venous return, *Slope*_*vrc*_ slope of venous return curve, *P*_*msf*_ mean systemic filling pressure, *R*_*vo*_ resistance for ventricular output, *Slope*_*voc*_ slope of ventricular output curve, *P*_*cc*_ critical closing pressure, *BFV*_*mca*_ blood flow velocity in middle cerebral artery, *RBC* red blood cell, *PBR* perfused boundary region, *TVD* total vascular density, *PVD* perfused vascular density, *VVD* valid vascular density, *PV* perfused vessels

* *P* < 0.01; ** *P* < 0.001; *** *P* < 0.0001 for paired comparison between indicated outcome and baseline (T1)

^a^Directly measured from radial artery and central venous catheters

^b^Calculated beat-to-beat by pulse contour analysis from Modelflow and averaged over indicated time period

^c^Calculated by pulse contour analysis from LiDCO*plus* 15 min after starting the exposure at indicated time period

^d^Secondarily derived from Modelflow calculated variables

^e^Directly measured using transcranial Doppler on middle cerebral artery

^f^Calculated from sublingual sidestream dark field imaging analyses

**Fig. 1:**
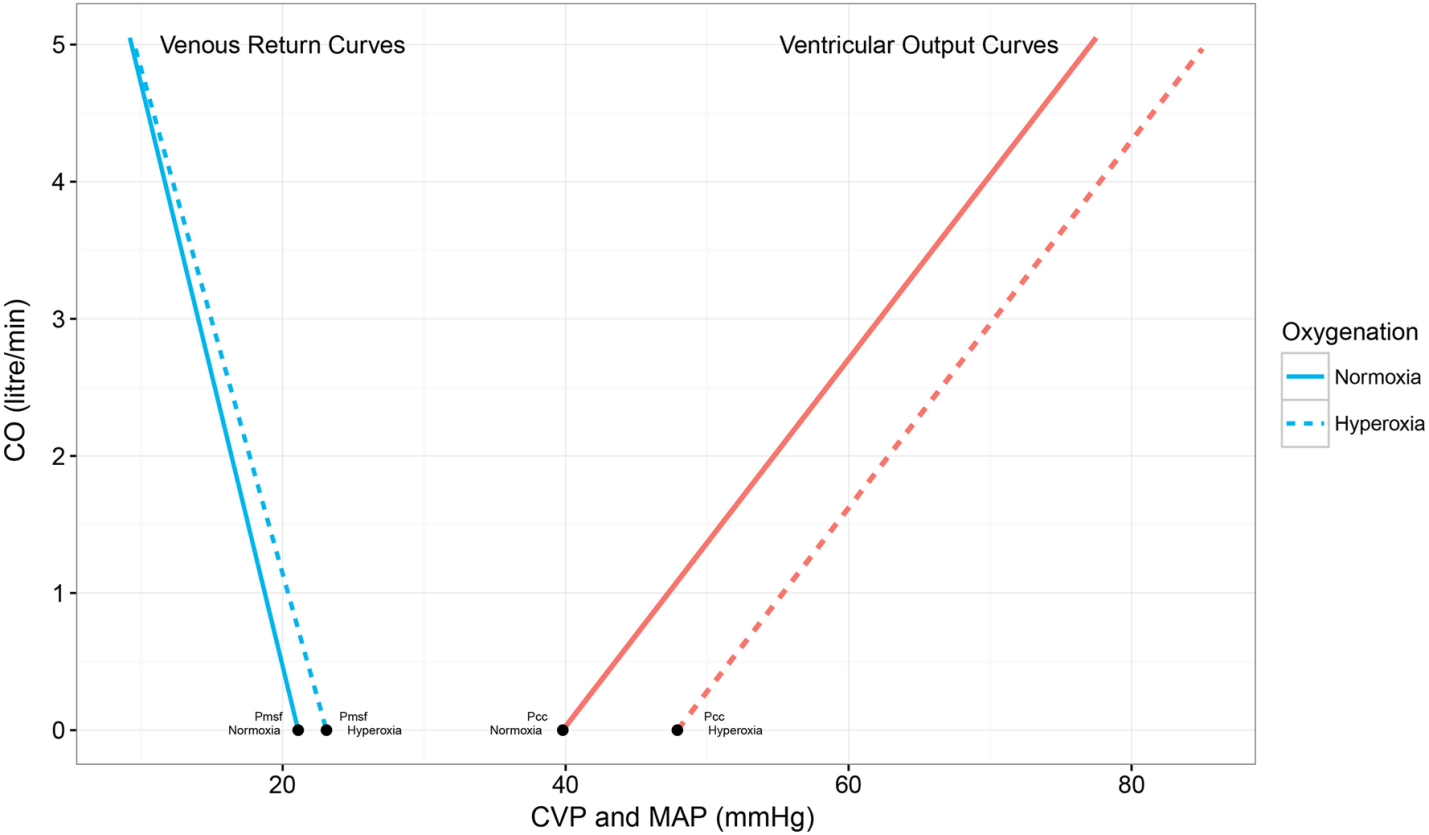
Venous return and ventricular output curves by arterial oxygenation status. Relationship between cardiac output (CO) and central venous pressure (CVP) in a venous return curve and between CO and mean arterial blood pressure (MAP) in a ventricular output curve for the averaged patient (Table 3). At zero blood flow mean systemic filling pressure (*P*_msf_) and critical closing pressure (*P*_cc_) are indicated. Venous return curves and ventricular output curves for arterial normoxia and hyperoxia are given

During the hyperoxia period *P*_msf_ and the slope of the venous return curve (Slope_vrc_) increased (Fig. 1). *P*_cc_ increased, whereas the slope of the left ventricular output curve (Slope_voc_) did not change. *R*_sys_ and *R*_vr_ increased because of the higher MAP and *P*_msf_ at constant CO. *R*_vo_ did not change because MAP and *P*_cc_ increased similarly.

We did not find any reduction in cerebral blood flow and only slight shifts in microcirculatory scores were noted. The percentage of perfused vessels decreased during hyperoxia (*P* = 0.01). No changes in vascular density were detected for either large or small vessels.

The results were virtually unchanged when multivariate mixed models were used (Table 3).

## Discussion

In this single-center physiological intervention study, we found that a 15-min exposure to hyperoxia affects hemodynamics in ICU patients after CABG. This was translated in several changes in central circulatory variables and in the percentage of perfused microcirculatory vessels, but showed no alterations in cardiac output and cerebral blood flow.

The circulation of blood can be described by either the CO or the venous return. As only blood returning to the heart can be pumped out in the systemic circulation, venous return should always equal CO. Major determinants of CO are preload, contractility and afterload. During hyperoxia, left ventricular afterload clearly increased. The absence of a measurable decrease in CO may be explained by a concomitant increase in preload. Indeed, we found higher CVP during hyperoxia. The alternative explanation, i.e., increased contractility, is unlikely as we found no increase in the slope of the cardiac output curve during hyperoxia.

The circulation can also be described by the venous return to the heart, which is driven by *P*_msf_ − CVP. During hyperoxia *P*_msf_ increased more than CVP. However, this did not lead to an increase of venous return due to the simultaneous increase in venous resistance.

Vasoconstriction may be the key driver of most if not all effects of hyperoxia on hemodynamics. Not only does it increase blood pressure, afterload and venous resistance, it also leads to a shift of blood from unstressed to stressed volume, as indicated by increases in *P*_msf_ and CVP. We also observed an increase in *P*_cc_ by hyperoxia. *P*_cc_ is a theoretical pressure defined by dynamic pressure flow-relations and represents the arterial pressure, below which conceptually no flow will be possible. It is a combined estimate representing all vascular circuits. Theoretically, *P*_cc_ is the sum of arterial wall tension and the pressure surrounding the blood vessel. *P*_cc_ may differ importantly between different vessels, and measured *P*_cc_ is an average value for the complete vasculature. An increase in *P*_cc_ may be especially relevant in certain disease states such as increased intracranial pressure and abdominal compartment syndrome with high pressures surrounding the vessels. In both situations, *P*_cc_ is markedly elevated. For example, intracerebral blood flow will decrease to zero when arterial blood pressure is lower than the critical closing pressure of the brain and beyond the limits of cerebral autoregulation. In such states, vasoconstriction, by either vasoactive drugs or hyperoxia, may have either beneficial effects by increasing blood pressure or harmful effects by increasing arterial wall tone and thereby *P*_cc_.

In our study, we could not show a reduction in perfusion of the brain by hyperoxia. Similarly, in a previous report perfusion changes at all oxygen levels were relatively small [27, 28]. It should be noted that all these studies were performed in situations with normal intracranial pressure. In situations with intracranial hypertension, such as in traumatic brain injury, we cannot rule out that a further increase of *P*_cc_ by hyperoxia will decrease the pressure difference between *P*_a_ and *P*_cc_ which can lead to a lower perfusion and possible ischemia of the brain.

Comparing our findings with earlier studies on the effects of hyperoxia on hemodynamics, the cardiac output fall was less than in healthy volunteers [29] and the decrease in the percentage of perfused vessels of the microcirculation was also in a different order of magnitude than previously observed [30]. Recognizing the perfused boundary region of microcirculatory vessels as a surrogate measure for the dimension of the glycocalyx, we could not detect any hyperoxia-induced alterations. Considering the effects on the venous system, more pronounced effects are to be expected in the smaller vessels compared to larger vessels. As a limited number of arterioles are present in the sublingual mucosa, where capillaries and venules are more abundant, only slight changes were anticipated in the analyzed microcirculation when high oxygen levels are applied.

Study differences may be largely explained by the use of anesthesia and mechanical ventilation as both affect hemodynamics and anesthesia also induces a considerable decrease in stressed volume. Furthermore, even in the presence of healthy lungs, both mechanical ventilation and bypass surgery may inflict an inflammatory response which can modify the effects of hyperoxic ventilation on the circulation in comparison with healthy subjects. Remaining differences may be clarified by the short exposure time in our procedures, although no further increase in PaO_2_ was to be expected from a longer exposure and therefore a steady state in hemodynamics was assumed.

The increase in stressed volume and *P*_msf_ by hyperoxia mimics the effects of administering a fluid bolus, yet without increasing the *R*_vr_. It is well known that the effects of extra fluids on CO are most pronounced in situations with underfilling of the vasculature explaining the relative conservation of cardiac output during hyperoxia in our postoperative, sedated patients, compared to healthy subjects. The effects of hyperoxia closely resemble the effects of norepinephrine and are in contrast to the effects of propofol [31, 32]. We earlier showed that intravenous administration of norepinephrine resulted in increases in *R*_sys_, *R*_vr_ and *P*_msf_. Interestingly, CO increased in some but not all patients after norepinephrine [32]. An increase in CO was associated with a higher SVV. Thus, it appears likely that the effects of a shift from unstressed to stressed volume by vasoconstriction, with an increase in *P*_msf_, is mostly found in patients with vasoplegia and/or a decreased circulating volume. Hence, the effects of hyperoxia on CO are determined by the balance between volume recruitment (*P*_msf_) and change in *R*_vr_ and baseline heart function, as observed before [32]. Although our results clearly indicate that hyperoxia increases venous resistance by venous vasoconstriction and that left ventricular output resistance (*R*_vo_) did not change, we must realize that our description of the circulation is not complete. We cannot describe the part between the site where *P*_cc_ exists and the site where *P*_msf_ exists. Therefore, there is a missing part of the circulatory circuit, i.e., the distal arterial compartment, where control of the peripheral circulation is performed by the pre-capillary sphincters.

A recent study with an alternative cardiac output monitor of the arterial pressure wave showed a poor correlation with the thermodilution obtained CO values while changing norepinephrine doses [33]. However, measurements carried out by our group suggest that the Modelflow technique is capable of measuring the effects induced by vasoconstriction in an accurate manner [32], suggesting that vasoactive agents may not importantly affect the precision of your technology. This was also underlined by the CO values measured by the LiDCO*plus* monitor that showed a similar pattern compared to the Modelflow technique in our study. Furthermore, the determination of *P*_msf_ is not dependent on the accuracy of the Modelflow technique. Indeed, extrapolation of the venous return curve to flow zero is independent of absolute cardiac output. The ability to follow changes in cardiac output within a patient has been clearly demonstrated before [18]. We also showed that beat-to-beat changes in Modelflow cardiac output follows cardiac output by beat-to-beat analysis of electromagnetic probe flow signals [19].

Acknowledging that our findings are to be reproduced in a larger cohort and different clinical settings, the following study aspects should be considered. First, the small sample size and the specific subgroup of patients do not warrant a broad generalizability for the observed effects. Hemodynamics in the current patient group may be affected by the effects of the recent bypass and the potential mediators of ischemia reperfusion and inflammation. Other subsets of critically ill patients may respond differently than patients in our cohort who were in a relatively stable condition before starting study procedures. Two patients received small doses of norepinephrine during the experiment to keep the blood pressure in the same order of magnitude as the other included patients but showed stable hemodynamics and the dose was not changed throughout the experiment. Also, excluding these patients from our analyses showed virtually no change in our results.

There may be a time effect in which recovery and stabilization of patients in the ICU after surgery may influence hemodynamics. However, assuming that the effect of hyperoxia was transient and respecting a 15-min time gap between the two exposures, the carryover effect was minimized and each case served as its own control (self-matched) [34]. Adjusted changes in estimates were based on within-subject comparisons of exposure to hyperoxia with exposure to normoxia. Sampling bias was minimized by continuously measuring central circulatory variables, which provide a highly accurate representation of the parameters over the time periods. Cerebral blood flow, microcirculation and parameters assessed from the inspiratory hold procedures were measured intermittently, yet at representative sampling moments during the sequential time points and averaged as appropriate.

Since we could not detect large differences between the outcomes of univariate and multivariate statistical models accounting for repeated measurements, the observed effects may be predominantly attributed to hyperoxic ventilation, rather than to concomitant changes in other parameters. Other covariates that were considered, such as PaCO_2_, are therefore not a likely explanation for the hemodynamic changes as seen during the experiment. While a short period of supraphysiological arterial oxygenation may disturb the hemodynamic balance, the effects of long-term exposure to hyperoxia are still uncertain but may be essential regarding patient-centered outcomes.

## Conclusions

Short-term hyperoxia after cardiac surgery induces significant alterations in systemic circulation mainly by vasoconstriction of both the venous and arterial circulation and an increase of mean systemic filling pressure. The increase in stressed volume and systemic filling pressure by hyperoxia resembles the effects of administering a fluid bolus or norepinephrine. This may have clinically important consequences in critically ill patients when hemodynamic and microcirculatory changes are vital, but the effects were not clearly linked to relevant changes in cardiac output and cerebral blood flow.

## Abbreviations

APACHE: Acute Physiology and Chronic Health Evaluation Score
APRV: airway pressure release ventilation
AVA: Automated Vascular Analysis
BFV: blood flow velocity
CABG: coronary artery bypass grafting
CO: cardiac output
CVP: central venous pressure
ICU: intensive care unit
FiO_2_: fraction of inspired oxygen
Hb: hemoglobin
Ht: hematocrit
HR: heart rate
MAP: mean arterial pressure
MCA: middle cerebral artery
NTR: Netherlands Trial Register
PaCO_2_: partial pressure of arterial carbon dioxide
PaO_2_: partial pressure of arterial oxygen
PBR: perfused boundary region
*P*_cc_: critical closing pressure
PEEP: positive end-expiratory pressure
*P*_insp_: inspiratory pressure
*P*_msf_: mean systemic filling pressure
PPV: pulse pressure variation
PV: perfused vessels
PVD: perfused vessel density
RBC: red blood cell
*R*_sys_: resistance of the systemic circulation
*R*_vo_: resistance for ventricular output
*R*_vr_: resistance for venous return
SaO_2_: arterial oxygen saturation
SAPS: Simplified Acute Physiology Score
SDF: sidestream dark field
Slope_voc_: slope of ventricular output curve
Slope_vrc_: slope of venous return curve
SVV: stroke volume variation
TCD: transcranial Doppler
TVD: total vessel density
*V*_T_: tidal volume
VVD: valid vessel density

## References

[1] Rachmale S, Li G, Wilson G, Malinchoc M, Gajic O (2012). Practice of excessive F(IO(2)) and effect on pulmonary outcomes in mechanically ventilated patients with acute lung injury. Respir Care.

[2] Helmerhorst HJ, Schultz MJ, van der Voort PH, de Jonge E, van Westerloo DJ (2015). Bench-to-bedside review: the effects of hyperoxia during critical illness. Crit Care.

[3] Sinclair SE, Altemeier WA, Matute-Bello G, Chi E (2004). Augmented lung injury due to interaction between hyperoxia and mechanical ventilation. Crit Care Med.

[4] Li LF, Liao SK, Ko YS, Lee CH, Quinn DA (2007). Hyperoxia increases ventilator-induced lung injury via mitogen-activated protein kinases: a prospective, controlled animal experiment. Crit Care.

[5] Helmerhorst HJ, Roos-Blom MJ, van Westerloo DJ, de Jonge E (2015). Association between arterial hyperoxia and outcome in subsets of critical illness: a systematic review, meta-analysis, and meta-regression of cohort studies. Crit Care Med.

[6] Farquhar H, Weatherall M, Wijesinghe M, Perrin K, Ranchord A, Simmonds M, Beasley R (2009). Systematic review of studies of the effect of hyperoxia on coronary blood flow. Am Heart J.

[7] Rousseau A, Bak Z, Janerot-Sjoberg B, Sjoberg F (2005). Acute hyperoxaemia-induced effects on regional blood flow, oxygen consumption and central circulation in man. Acta Physiol Scand.

[8] Kilgannon JH, Jones AE, Shapiro NI, Angelos MG, Milcarek B, Hunter K, Parrillo JE, Trzeciak S, Emergency Medicine Shock Research Network (EMShockNet) Investigators (2010). EMSRN: association between arterial hyperoxia following resuscitation from cardiac arrest and in-hospital mortality. JAMA.

[9] Wang CH, Chang WT, Huang CH, Tsai MS, Yu PH, Wang AY, Chen NC, Chen WJ (2014). The effect of hyperoxia on survival following adult cardiac arrest: a systematic review and meta-analysis of observational studies. Resuscitation.

[10] Davis DP, Meade W, Sise MJ, Kennedy F, Simon F, Tominaga G, Steele J, Coimbra R (2009). Both hypoxemia and extreme hyperoxemia may be detrimental in patients with severe traumatic brain injury. J Neurotrauma.

[11] Rincon F, Kang J, Maltenfort M, Vibbert M, Urtecho J, Athar MK, Jallo J, Pineda CC, Tzeng D, McBride W (2014). Association between hyperoxia and mortality after stroke: a multicenter cohort study. Crit Care Med.

[12] Rincon F, Kang J, Vibbert M, Urtecho J, Athar MK, Jallo J (2014). Significance of arterial hyperoxia and relationship with case fatality in traumatic brain injury: a multicentre cohort study. J Neurol Neurosurg Psychiatry.

[13] de Jonge E, Peelen L, Keijzers PJ, Joore H, de Lange D, van der Voort PH, Bosman RJ, de Waal RA, Wesselink R, de Keizer NF (2008). Association between administered oxygen, arterial partial oxygen pressure and mortality in mechanically ventilated intensive care unit patients. Crit Care.

[14] Stub D, Smith K, Bernard S, Nehme Z, Stephenson M, Bray JE, Cameron P, Barger B, Ellims AH, Taylor AJ (2015). Air versus oxygen in ST-segment-elevation myocardial infarction. Circulation.

[15] McGuinness SP, Parke RL, Drummond K, Willcox T, Bailey M, Kruger C, Baker M, Cowdrey KA, Gilder E, McCarthy L (2016). A multicenter, randomized, controlled phase IIb trial of avoidance of hyperoxemia during cardiopulmonary bypass. Anesthesiology.

[16] Smit B, Smulders YM, de Waard MC, Boer C, Vonk AB, Veerhoek D, Kamminga S, de Grooth HJ, Garcia-Vallejo JJ, Musters RJ (2016). Moderate hyperoxic versus near-physiological oxygen targets during and after coronary artery bypass surgery: a randomised controlled trial. Crit Care.

[17] De Backer D, Ortiz JA, Salgado D (2010). Coupling microcirculation to systemic hemodynamics. Curr Opin Crit Care.

[18] Jansen JR, Schreuder JJ, Mulier JP, Smith NT, Settels JJ, Wesseling KH (2001). A comparison of cardiac output derived from the arterial pressure wave against thermodilution in cardiac surgery patients. Br J Anaesth.

[19] Maas JJ, Geerts BF, Jansen JR (2011). Evaluation of mean systemic filling pressure from pulse contour cardiac output and central venous pressure. J Clin Monit Comput.

[20] Maas JJ, Geerts BF, van den Berg PC, Pinsky MR, Jansen JR (2009). Assessment of venous return curve and mean systemic filling pressure in postoperative cardiac surgery patients. Crit Care Med.

[21] De Backer D, Hollenberg S, Boerma C, Goedhart P, Buchele G, Ospina-Tascon G, Dobbe I, Ince C (2007). How to evaluate the microcirculation: report of a round table conference. Crit Care.

[22] Lee DH, Dane MJ, van den Berg BM, Boels MG, van Teeffelen JW, de Mutsert R, den Heijer M, Rosendaal FR, van der Vlag J, van Zonneveld AJ (2014). Deeper penetration of erythrocytes into the endothelial glycocalyx is associated with impaired microvascular perfusion. PLoS ONE.

[23] Vlahu CA, Lemkes BA, Struijk DG, Koopman MG, Krediet RT, Vink H (2012). Damage of the endothelial glycocalyx in dialysis patients. J Am Soc Nephrol.

[24] Donati A, Damiani E, Domizi R, Romano R, Adrario E, Pelaia P, Ince C, Singer M (2013). Alteration of the sublingual microvascular glycocalyx in critically ill patients. Microvasc Res.

[25] De Backer D, Donadello K, Sakr Y, Ospina-Tascon G, Salgado D, Scolletta S, Vincent JL (2013). Microcirculatory alterations in patients with severe sepsis: impact of time of assessment and relationship with outcome. Crit Care Med.

[26] Maas JJ, de Wilde RB, Aarts LP, Pinsky MR, Jansen JR (2012). Determination of vascular waterfall phenomenon by bedside measurement of mean systemic filling pressure and critical closing pressure in the intensive care unit. Anesth Analg.

[27] Bulte DP, Chiarelli PA, Wise RG, Jezzard P (2007). Cerebral perfusion response to hyperoxia. J Cereb Blood Flow Metab.

[28] Borzage MT, Bush AM, Choi S, Nederveen AJ, Vaclavu L, Coates TD, Wood JC (2016). Predictors of cerebral blood flow in patients with and without anemia. J Appl Physiol (1985).

[29] Bak Z, Sjoberg F, Rousseau A, Steinvall I, Janerot-Sjoberg B (2007). Human cardiovascular dose-response to supplemental oxygen. Acta Physiol (Oxf).

[30] Orbegozo Cortes D, Puflea F, Donadello K, Taccone FS, Gottin L, Creteur J, Vincent JL, De Backer D (2015). Normobaric hyperoxia alters the microcirculation in healthy volunteers. Microvasc Res.

[31] de Wit F, van Vliet AL, de Wilde RB, Jansen JR, Vuyk J, Aarts LP, de Jonge E, Veelo DP, Geerts BF (2016). The effect of propofol on haemodynamics: cardiac output, venous return, mean systemic filling pressure, and vascular resistances. Br J Anaesth.

[32] Maas JJ, Pinsky MR, de Wilde RB, de Jonge E, Jansen JR (2013). Cardiac output response to norepinephrine in postoperative cardiac surgery patients: interpretation with venous return and cardiac function curves. Crit Care Med.

[33] Monnet X, Anguel N, Jozwiak M, Richard C, Teboul JL (2012). Third-generation FloTrac/Vigileo does not reliably track changes in cardiac output induced by norepinephrine in critically ill patients. Br J Anaesth.

[34] Maclure M (1991). The case-crossover design: a method for studying transient effects on the risk of acute events. Am J Epidemiol.

